# Neuromagnetic Abnormality of Motor Cortical Activation and Phases of Headache Attacks in Childhood Migraine

**DOI:** 10.1371/journal.pone.0083669

**Published:** 2013-12-27

**Authors:** Jing Xiang, Xinyao deGrauw, Abraham M. Korman, Janelle R. Allen, Hope L. O'Brien, Marielle A. Kabbouche, Scott W. Powers, Andrew D. Hershey

**Affiliations:** 1 Division of Neurology, Cincinnati Children's Hospital Medical Center, Cincinnati, Ohio, United States of America; 2 Department of Pediatrics, University of Cincinnati, College of Medicine, Cincinnati, Ohio, United States of America; 3 Division of Behavioral Medicine and Clinical Psychology, Cincinnati Children's Hospital Medical Center, Cincinnati, Ohio, United States of America; Johns Hopkins School of Medicine, United States of America

## Abstract

The cerebral cortex serves a primary role in the pathogenesis of migraine. This aberrant brain activation in migraine can be noninvasively detected with magnetoencephalography (MEG). The objective of this study was to investigate the differences in motor cortical activation between attacks (ictal) and pain free intervals (interictal) in children and adolescents with migraine using both low- and high-frequency neuromagnetic signals. Thirty subjects with an acute migraine and 30 subjects with a history of migraine, while pain free, were compared to age- and gender-matched controls using MEG. Motor cortical activation was elicited by a standardized, validated finger-tapping task. Low-frequency brain activation (1∼50 Hz) was analyzed with waveform measurements and high-frequency oscillations (65–150 Hz) were analyzed with wavelet-based beamforming. MEG waveforms showed that the ictal latency of low-frequency brain activation was significantly delayed as compared with controls, while the interictal latency of brain activation was similar to that of controls. The ictal amplitude of low-frequency brain activation was significantly increased as compared with controls, while the interictal amplitude of brain activation was similar to that of controls. The ictal source power of high-frequency oscillations was significantly stronger than that of the controls, while the interictal source power of high-frequency oscillations was significantly weaker than that of controls. The results suggest that aberrant low-frequency brain activation in migraine during a headache attack returned to normal interictally. However, high-frequency oscillations changed from ictal hyper-activation to interictal hypo-activation. Noninvasive assessment of cortical abnormality in migraine with MEG opens a new window for developing novel therapeutic strategies for childhood migraine by maintaining a balanced cortical excitability.

## Introduction

Previous studies of migraine have suggested that not only can untreated or ineffectively treated migraines become progressive, but over the long-term, they may cause neurological changes significant enough to be quantified with neuroimaging [1]–[6]. The cerebral cortex serves a primary role in the pathogenesis of migraine. There is accumulating evidence that adults with migraine are associated with aberrant activation in the somatosensory, visual, and auditory cortices during attacks (ictal), as well as, during the pain free period (interictal) [7]–[10].

The involvement of the motor cortex in hemiplegic migraine, a small subset of migraine cases, is clinically significant [11]–[15]. With an alternate finger tapping task, psychomotor dysfunction has also been found in typical migraine diagnosed according to international headache society (IHS) criteria [16]. It seems that motor coordination is also impaired in patients with migraine during headache attacks [17]. Reports on non-familial migraine with unilateral motor symptoms (MUMS) showed that a syndrome of severe migraine with accompanying give-way weakness is common in tertiary care headache centers [5]. Approximately, 58% of patients with MUMS reported persistent weakness between headache attacks [5]. Functional magnetic resonance imaging (fMRI) study of migraine has found that the supplementary motor area (SMA) is abnormal in migraine even at resting state [18]. An increasing list of transcranial magnetic stimulation (TMS) reports indicate that motor cortical dysfunction may play an important role in the pathogenesis of attacks of migraine [19]–[24]. Importantly, high-frequency repetitive TSM (rTMS) of the motor cortex can normalize aberrant intracortical inhibition in migraine [25]. Neurophysiologically, rTMS of the motor cortex can also modulate pain-related evoked responses in migraine patients [26]. Recent reports have also revealed that the spread of abnormal ictal brain activation triggered by movements plays a key role in the pathogenesis of pediatric migraine [27]–[29]. It remains unclear whether these changes in motor cortical activation persist during the headache free period.

The development of neuroimaging technologies, such as magnetoencephalography (MEG) has made it possible to noninvasively investigate the underlying neurophysiological mechanisms of migraine [10], [30], [31]. It has been shown that neuromagnetic brain activation is significantly increased in patients with migraine [10], [32], [33]. Previous MEG studies of migraine typically focused on neuromagnetic waveforms in a low-frequency range, such as DC-MEG signals [10], [30], [31]. Recent reports suggest that the brain generates high-frequency oscillations (HFOs) or high-gamma oscillations that can be detected and localized with newly developed MEG methods such as wavelet-based beamforming techniques [29], [34]. The examination of HFOs has the potential to provide key information about the cerebral mechanisms of migraine, as HFOs are well-localized and can be quantified at source space [29]. Currently, no reports specifically focus on HFOs during interictal periods, or the correlations between HFOs and the phases of headache attacks in pediatric migraine. HFOs are important in the study of migraine for at least two reasons: (1) recent reports showed that migraine is associated with cortical hyper-excitability or hypo-excitability in various brain areas [23], [35]–[38]. HFOs, which can be well localized and quantified at source space, can provide precise information about where and to what degrees of alteration of cortical excitability is occurring in migraine. (2) Transcranial magnetic stimulation (TMS) [23], [35], [37], [39], [40] and other spatially targeted treatment (e.g. transcranial direct current stimulation, tDCS) [41] can reduce headache in migraine by normalizing focal cortical excitability. HFOs may provide critical spatial information to guide spatially targeted treatments for better clinical outcomes.

The aim of this study was to quantitatively determine if there are any differences in low- and high-frequency brain signals during ictal and interictal time periods using MEG. Since the pain of many migraine sufferers worsens with physical activity [42], [43] and previous reports have confirmed that neuromagnetic signals in 65–150 Hz (high-gamma oscillations) in motor cortex can be reliably elicited by a finger tapping task [34], [44], [45], this study focused on neuromagnetic high-gamma oscillations in the motor cortex. MEG data were analyzed with the conventional waveform measurement [46], as well as, a new wavelet based beamforming technique [47]. The new technique enabled us to quantify neuromagnetic high-gamma oscillations at source space [28], while the conventional waveform measurements enabled us to analyze low-frequency brain activation and compare our results with previous reports typically focusing on MEG waveforms [10], [48].

## Materials and Methods

### Participants

Sixty patients with migraine (migraine subjects) were recruited from the Headache Center at Cincinnati Children's Hospital Medical Center (CCHMC). Twenty eight ictal subjects (20 girls, 8 boys; mean age ±SD: 15.0±2.1 years) and 28 interictal subjects (20 girls, 8 boys; mean age ±SD: 15.3±2.3 years) were analyzed. Of the 60 subjects, 4 subjects did not meet the inclusion and exclusion criteria. Inclusion criteria were migraine without aura as defined by the International Classification of Headache Disorders, 2nd Edition (ICHD-II) [49], [50]; and no other neurological disorder. Interictal subjects were recorded at least 3 days before or after a migraine attack. Healthy controls were recruited to match the patients for age and gender and met inclusion criteria of being healthy without a history of neurological disorders, migraine, or brain injury, and age-appropriate hearing, vision, and hand movement. Exclusion criteria for all participants were: (1) presence of an implant, such as cochlear implant devices, a pacemaker or neuro-stimulator, devices containing electrical circuitry, generating magnetic signals, or having other metal that could produce visible magnetic noise in the MEG data; (2) inability to remain still; (3) inability to cooperate with personnel operating the MEG equipment. The research protocol, assent and consent forms were formally reviewed and approved by the Institutional Review Board (IRB) at CCHMC. The migraine subjects were pre-screened by neurologists certified in headache medicine. If a subject and parent/guardian met the criteria and were interested in our MEG study, a researcher would explain the research protocol and obtain written informed assent and consent from the participant and her/his parents. Both the participant and her/his parents were provided with a questionnaire that included an assessment of headache (pain) severity on a scale of 0 to 10 (10 being the worst). The MEG recordings for ictal subjects were performed prior to initiation of treatment. The MEG recordings for interictal subjects were performed after confirming that the subjects did not have migraine attacks for at least 3 days.

### Motor Task

All subjects performed a brisk index finger tapping task with either the right or the left index finger immediately after hearing a cue (500 Hz, square wave tone). Subjects were instructed to press a response button with the index finger that was ipsilateral to the tone presented, while keeping other body parts still [34]. Their eyes were open and fixed to an arbitrary target during the paradigm. A trigger was sent to the MEG system from the response box when the button was pressed. The stimuli consisted of 200 trials of square tones, 100 trials per ear, and were presented randomly through a plastic tube and earphones. The inter-stimulus interval of the sound cue was 0–1000 ms, which varied from 0 to 1000 ms randomly. Stimulus presentation and response recording were accomplished with BrainX software, which was based on DirectX (Microsoft Corporation, Redmond, WA, USA) [34].

### MEG Recordings

The MEG signals were recorded in a magnetically shielded room (Vacuum-Schmelze, Hanau, Germany) using a whole-cortex CTF 275-Channel MEG system (VSM MedTech Systems Inc., Coquitlam, BC, Canada). Before data acquisition began, electromagnetic coils were attached to the nasion, left and right pre-auricular points of each participant. These three coils were subsequently activated at different frequencies for measuring participants' head positions relative to the MEG sensors. The sampling rate of the MEG recordings was 6000 Hz (very high-frequency signals were analyzed in another study). An acquisition window was set to 3000 milliseconds (ms) per trial, with 2000 ms pre-trigger. Data were recorded with a noise cancellation of third order gradients. Subjects were asked to remain still. If head movement during a recording was beyond 5 mm, that dataset was indicated as “bad” and an additional trial was recorded.

### Magnetic Resonance Imaging (MRI) Scans

Three-dimensional (3D) MRI was obtained using a 3 T Philips Achieva (Philips Healthcare, 3000 Minuteman Road, Andover, MA). Three fiduciary points were placed in identical locations to the positions of the three coils used in the MEG recordings, with the aid of digital photographs to allow for an accurate co-registration of the two data sets. Subsequently, all anatomical landmarks were made identifiable in the MRIs. Pediatric Brain Templates developed by the Pediatric Neuroimaging Research Consortium (PNRC) [51] and CCHMC MEG Center [34] were also used for group comparison and visualization.

### Waveform Analyses

To analyze conventional low-frequency brain activity [8], [29], [52], [53], MEG waveforms were manually averaged using MEG Processor for identification of response components (deflections). The averaged MEG data were preprocessed by removing the DC offset based on the pre-trigger baseline as well as linear trend. The triggers were elicited by pressing the response button. An off-line high pass filter and low pass filter were applied for viewing signals in 1–50 Hz. The latencies and amplitudes of each recognizable peak were measured for each subject with a customer-designed program, MEG Processor.

### Wavelet-based Beamforming Analyses

Magnetic sources were scanned with a newly developed beamforming technique [47]. Each voxel in the source scan utilized a sensor beam according to its lead field. Multiple local spheres were used for magnetic forward computing. MEG Processor was used to compute magnetic sources [45]. Before doing beamforming analysis, a multiple local sphere head model was created for each subject. Beamforming was applied to estimate the cortical source power integrated over the time window for 65–150 Hz frequency band in 5 mm steps. The time-window and frequency band were determined by using our pilot data, as well as, normative data from previous experiments [29], [45]. The choosing of 65–150 Hz also allowed us to avoid the power-line noise around 60 Hz. The results were visualized using a Magnetic Source Locator (MSL) software program [29], [45].

### Statistical Analyses

MEG measurements were statistically analyzed with multiple analyses of variance (ANOVA). The fixed factors were group (interictal, ictal, and control groups) and age (categorized by quartiles). The dependent variables were magnetic source power and the latency and amplitude of waveforms. For multiple comparisons, a Bonferroni multiple comparisons correction was applied. The odds ratio of activity in brain areas among the migraine and control groups was analyzed with Fisher's exact tests. The correlation between headache severity and MEG parameters (latency, amplitude, and magnetic source power) were analyzed with spearman correlation. Significance was accepted at the level of p<0.05 for one test. If multiple testing were to be taken into account then the significance level for any one of these tests were reduced from 0.05 to 0.025 (two parameters) or 0.016 (three parameters).

## Results

### 1. Clinical Characteristics

Twenty out of the 28 ictal subjects and 20 out of the 28 interictal subjects were girls (20/28, 71%). The gender ratio in the ictal and interictal groups was 2.5 vs. 1.0. In the ictal group, 24 subjects had moderate to severe headache (24/28, 86%) and 22 subjects had bilateral headache attacks (22/28, 78%). The severity (scale from 0 to 10) of headache attack's range (mean ±SD) in the migraine subjects during headache attacks was 6.8±2.7.

### 2. Low-frequency Waveforms

**Figure 1:**
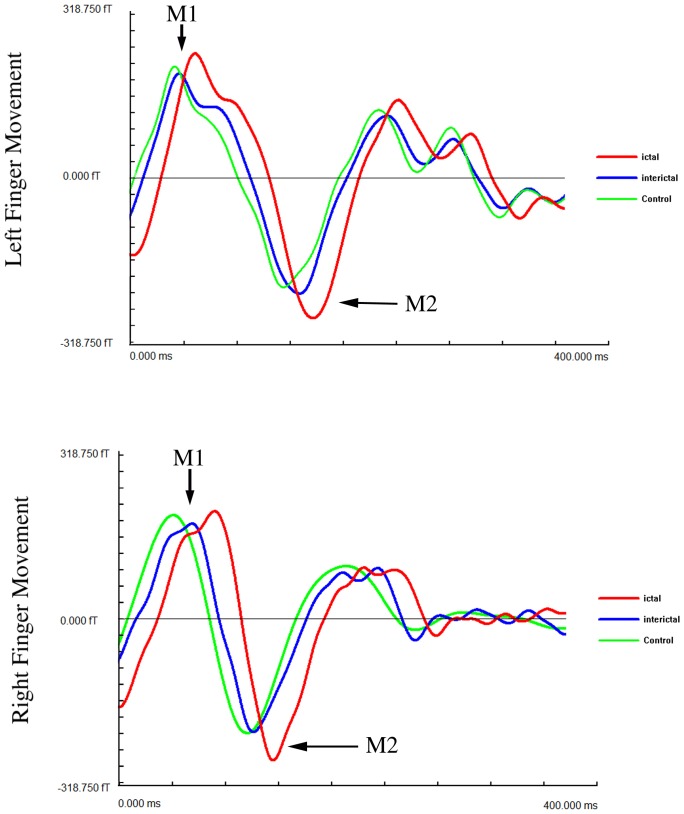
Movement-evoked magnetic waveforms from a subject during a migraine attack (“Ictal”), a subject between migraine attacks (“Interictal”), and a healthy control (“Control”). One waveform is from one sensor with highest amplitude of M1 among all sensors. Two neuromagnetic responses (“M1” and “M2”) following left or right finger movements are identifiable on each waveform in all the three subjects. The X-axis indicates latency; the Y-axis indicates amplitude.

**Figure 2 pone-0083669-g002:**
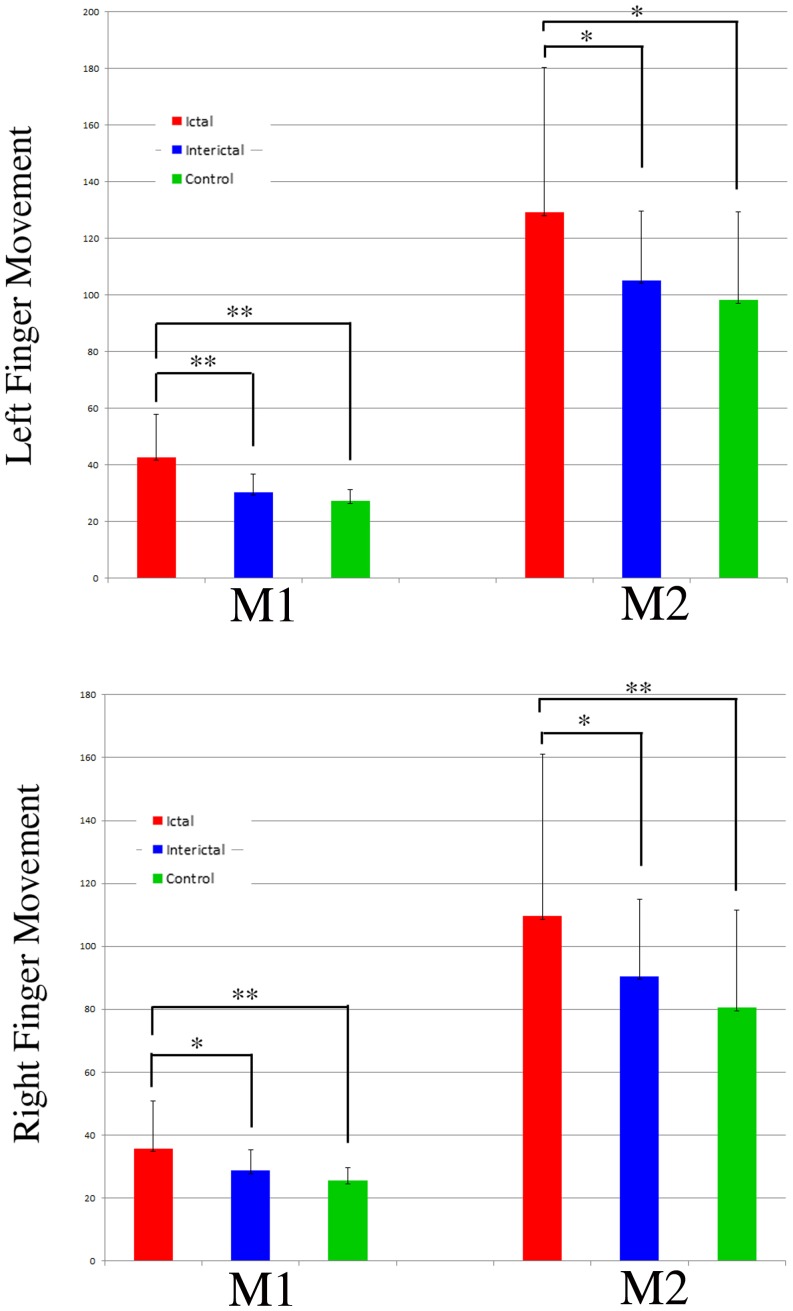
The latencies of the first two movement-evoked magnetic responses (“M1” and “M2”) during a migraine attack (“Ictal”), between attacks (“Interictal”), and healthy control (“Control”). Each bar represents the mean value and standard deviation (SD) of the corresponding latency. “**” indicates p<0.01; “*” indicates p<0.025.

**Figure 3:**
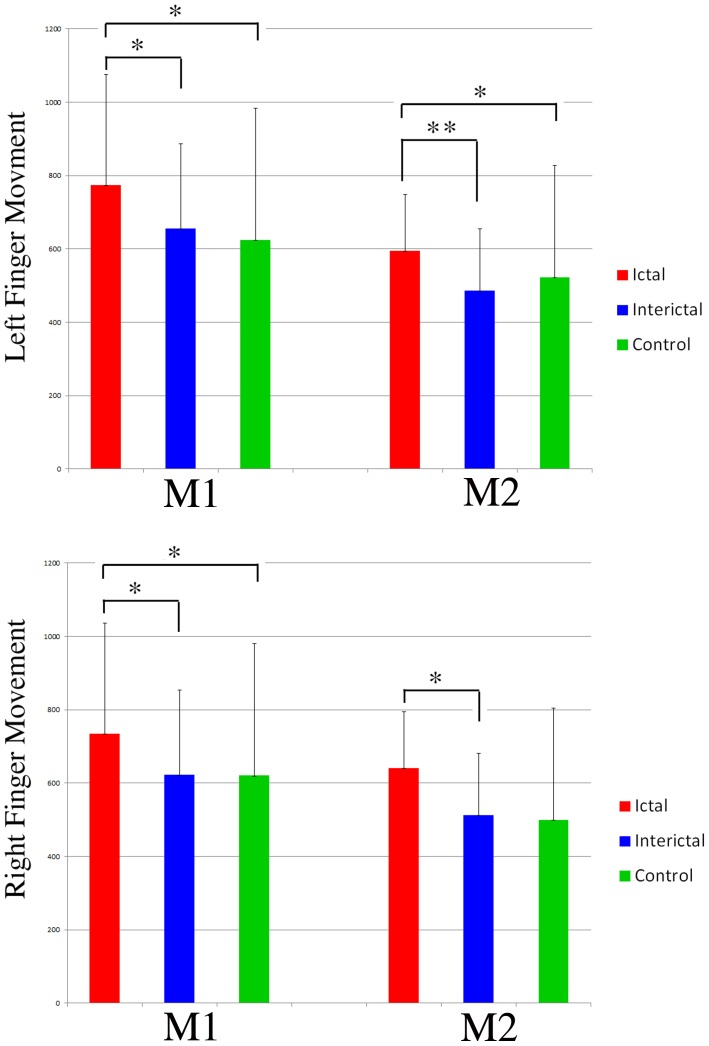
The amplitudes of the first two movement-evoked magnetic responses (“M1” and “M2”) during a migraine attack (“Ictal”), between attacks (“Interictal”), and healthy control (“Control”). Each bar represents the mean value and standard deviation (SD) of the corresponding amplitude. “*” indicates p<0.05; “**” indicates p<0.025.

The MEG waveforms in 1–50 Hz recorded from 24 out of the 28 ictal subjects and 24 out of the 28 interictal subjects showed at least two responses (deflections), which were named as M1 and M2. All the age- and gender-matched healthy controls had two responses. Figure 1 shows representative waveforms from a subject during a migraine attack (ictal), a subject between migraine attacks (interictal), and a control. Since the waveforms were elicited by left or right finger movements, there were two sets of waveforms for each group of subjects. Both ictal and interictal MEG waveforms had a larger variation in morphology among subjects in each group than control MEG waveforms. The latencies and amplitudes of interictal MEG waveforms were more similar to those of the control waveforms than to those of the ictal MEG waveforms. The quantitative measurements of the waveforms of all the three groups of subjects are shown in Figure 2 and Figure 3. To be consistent, in Figures 1–3, red indicates ictal data, blue indicates interictal data, and green indicates control data.

ANOVA with repeated measures revealed that headache attacks significantly affected the latencies of M1 and M2 (Figure 2), independent of age and gender (F = 7.94, p<0.001). Pairwise comparisons found that there were significant latency differences of M1 and M2 between ictal and control groups during left (p<0.01; p<0.025) and right (p<0.01; p<0.01) finger movements, respectively.

There were no latency differences of M1 and M2 between interictal and control groups following left or right finger movements (p>0.05). Compared with ictal group, the latencies of M1 and M2 in interictal group were significantly shortened during left (p<0.01; p<0.025) or right (p<0.025, p<0.025) finger movements, respectively. In other words, the interictal latencies of M1 and M2 were similar to the controls and were significantly different from ictal latencies.

ANOVA with repeated measures revealed that headache attacks significantly affected the amplitudes of M1 and M2 (Figure 3). The amplitude of M2 during left finger movement was mostly affected (F = 4.86, p<0.025), independent of age and gender. Pairwise comparisons revealed a significant amplitude difference of M1 between ictal and control groups during left or right finger movement (p<0.05). Though the latency of ictal M2 during right finger movement was longer than that of the control, there was no statistical difference.

There were no amplitude differences of M1 and M2 between interictal and control groups following left or right finger movements (p>0.05). Compared with ictal group, the amplitudes of M1 and M2 in interictal group were significantly decreased during left or right finger movements (p<0.05). Thus, the interictal amplitudes of M1 and M2 were similar to the controls and were significantly different from ictal amplitudes.

### 3. High-gamma Oscillations

**Figure 4:**
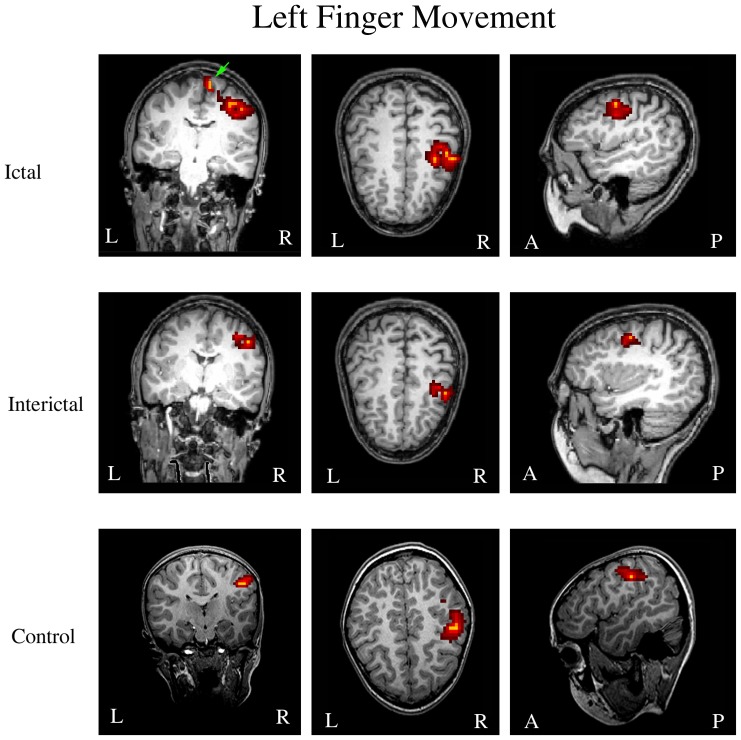
Magnetic source imaging (MSI) showing the locations of left finger movement-elicited high-gamma oscillations in a subject during a migraine attack (“Ictal”), a subject between attacks (“Interictal”), and a healthy control (“Control”). The primary motor cortex in the contralateral hemisphere is activated in all three subjects. The supplementary motor area is activated only during a migraine attack (green arrow). “R” indicates right; “L” indicates left. “A” indicates anterior; “P” indicates posterior.

**Figure 5:**
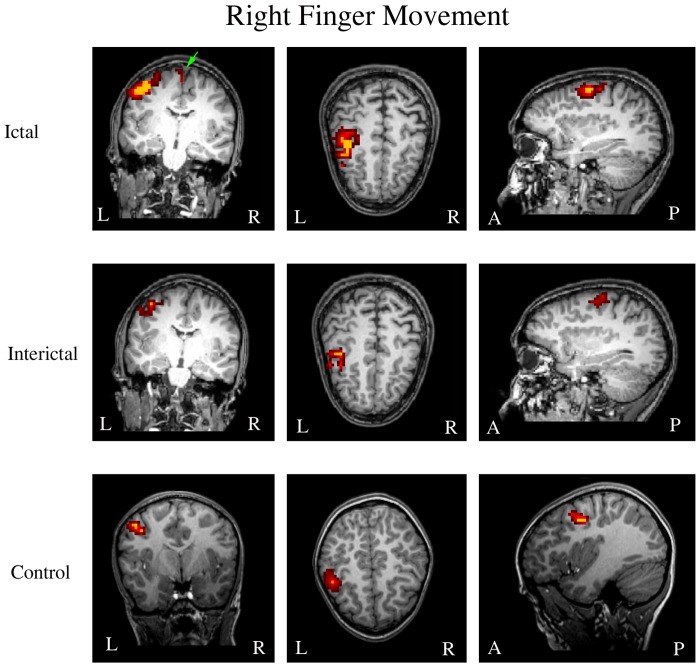
Magnetic source imaging (MSI) showing the locations of right finger movement-elicited high-gamma oscillations during a migraine attack (“Ictal”), between attacks (“Interictal”), and a healthy control (“Control”). The primary motor cortex in the contralateral hemisphere is activated in all three subjects. The supplementary motor area is activated only during a migraine attack (green arrow). “R” indicates right; “L” indicates left. “A” indicates anterior; “P” indicates posterior.

The MEG source imaging data were analyzed in an effort to determine the ictal and interictal spatial and spectral signatures of aberrant high-gamma oscillations (Figure 4 and Figure 5). The high-gamma oscillations were localized to the contralateral primary motor cortex in 28 ictal subjects and 28 interictal subjects. High-gamma oscillations in the 28 age- and gender-matched controls were also localized to the contralateral primary motor cortex. There was no significant difference among the three groups in terms of source location (X, Y, and Z coordinates) in the primary motor cortex (p>0.05). The MNI (Montreal Neurological Institute) coordinates of the location of movement-elicited brain activation are shown in Table 1. Though there were no statistical differences of the coordinates of the location of movement-elicited brain activation between the three groups of subjects, the ictal groups tended to have a greater variation among subjects within the group, which was indicated by a larger standard deviation.

**Table 1:** The MNI coordinates of the source locations of movement-elicited brain activation (in millimeters).

Moving Finger	Location	Ictal	Interictal	Control
**Left**	M1*	51.9±5.2$	49.8±4.6	48.2±3.8
	(28/28)#	−6.6±4.1	−5.4±3.9	−4.6±3.2
		49.7±4.9	50.4±4.1	50.4±3.8
	SMA*	1.9±8.1	1.7±5.6	1.4±4.9
	(24/28)	1.5±6.3	1.6±5.8	1.8±5.2
		51.2±7.6	52.7±6.4	53.3±6.6
**Right**	M1	−49.1±6.3	−47.8±4.6	−48.2±3.8
	(28/28)	−8.6±4.1	−7.6±3.9	−7.6±3.2
		51.7±4.9	50.8±4.1	50.4±3.8
	SMA	−2.1±6.1	−1.4±5.3	−1.7±4.2
	(24/28)	1.9±7.3	2.1±4.8	1.5±3.7
		52.2±8.6	51.7±6.2	50.1±5.3

*M1: primary motor cortex; SMA: supplementary motor area.

^#^ Number of subjects with activation/total number of tested subjects.

Mean ± Standard Deviation. The three numbers are the values of X, Y and Z coordinates.

High-gamma oscillations were identified in the supplement motor area (SMA) in 7 interictal subjects (25%, 7/28), 24 ictal subjects (86%, 24/28) and 6 healthy controls (23%, 6/26) following left or right finger movements. Ictal subjects had significantly higher odds of activation in the SMA (p<0.001) as compared to either healthy controls or interictal subjects. There was no significant difference between ictal subjects and healthy controls in terms of the activation in the SMA following either left or right finger movements (p>0.05).

Analyses of neuromagnetic source power in the primary motor cortex with ANOVA revealed that the strength of neuromagnetic activation was significantly affected by the headache attack phases (F = 6.792, p<0.01) following left or right finger movements (F = 7.864, p<0.005), independent of age and gender.

**Figure 6:**
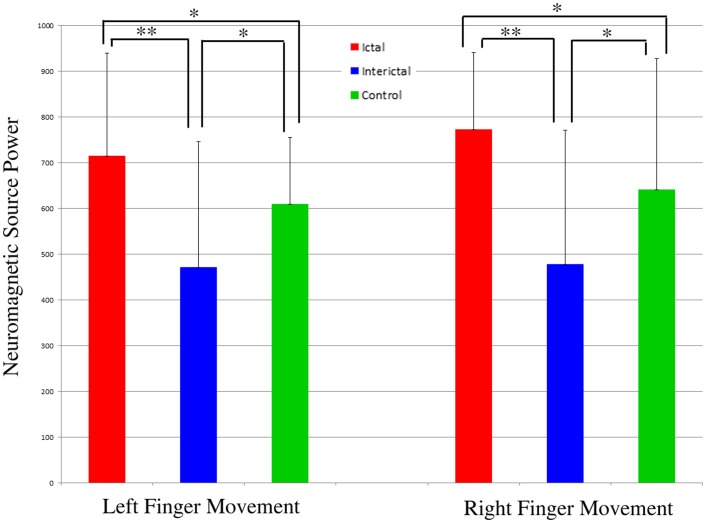
The neuromagnetic source power elicited by finger movements during a migraine attack (“Ictal”), between attacks (“Interictal”), and healthy control (“Control”). Each bar represents the mean value and standard deviation (SD) of the corresponding neuromagnetic source power. “**” indicates p<0.01; “*” indicates p<0.025.

Post-hoc pairwise comparisons indicated that the strength of neuromagnetic activation in ictal subjects was significantly higher than that of healthy controls following left or right finger movements (p<0.025). The strength of neuromagnetic activation in interictal subjects was significantly lower than that of healthy controls following left or right finger movements (p<0.01). The strength of neuromagnetic activation in interictal subjects was significantly lower than that of ictal subjects following left or right finger movements (p<0.001). Figure 6 shows the summary of neuromagnetic source power elicited by finger movements in the three groups of subjects.

### 4. Headache Severity and Neuromagnetic Brain Activity

The correlations between the severity of headache attacks and the latencies of M1 and M2 were 0.48 and 0.47 (p>0.025) for left and 0.51 and 0.49 (p>0.025) for right finger movements. The correlations between the severity of headache attacks and the amplitudes of M1 and M2 were 0.54 and 0.46 (p>0.025) for left and 0.52 and 0.42 (p>0.025) for right finger movements. The correlations between the severity of headache attacks and the source power of high-gamma oscillations in the primary motor cortex were 0.68 (p<0.01) for left and 0.64 (p<0.01) for right finger moments.

## Discussion

The present study examined neuromagnetic activation in a low-frequency range of 1–50 Hz and high-gamma activation in 65–150 Hz during headache attack and pain free periods using conventional measurements of waveforms, as well as, newly developed source localization methods [8], [29], [33]. The frequency band in which the MEG data were analyzed was determined with several considerations and pilot data. The waveform analysis was based on an averaging of multi-trial MEG data. Averaging keeps time or phase-locked signals while minimizing time-variable signals (such as random noise). Since low-frequency brain activity change slowly with time and high-frequency brain activity changes rapidly with time, averaging keeps low-frequency brain activity, while minimizing high-frequency brain activity. The mathematical reasoning is that a small natural variation in time among multiple trials may significantly change the phase of high-frequency signals but not the low-frequency signals. Therefore, averaging based waveform analysis can only be used to analyze low-frequency signals (1–50 Hz) in our movement-related studies. Beamformer, on the other hand, was developed to analyze non-time-locked signals, because it computes a covariance matrix of MEG data without averaging the waveforms. For a given time-window, the higher the number of oscillatory waveforms (higher frequency), the more stable the covariance matrix will be. Consequently, beamformer is suitable for analyzing rapid oscillatory activity, such as high-gamma activity (65–150 Hz), because the time window of movement-related brain activity is limited (<400 ms). We chose 1–50 Hz and 65–150 Hz, but not 40–100 Hz, because the power-line noise in the USA is 60 Hz. MEG is sensitive to magnetic noise generated by power-lines, therefore, our study avoided this noise. Building on our previous study [34] and pilot data, we found 1–50 Hz is suitable for waveform analysis and 65–150 Hz is suitable for beamformer analysis.

The results have demonstrated that the latencies of neuromagnetic responses evoked by finger tapping during the attacks, ictal neuromagnetic responses, were significantly delayed as compared with age- and gender-matched healthy controls. This observation is consistent with previous reports on childhood migraine [28], [29]. This is the first report showing that the latencies of ictal neuromagnetic responses following finger movements were also significantly delayed as compared with the latencies of interictal neuromagnetic responses as well as controls. There were no latency differences between interictal neuromagnetic responses and controls. It seemed that the latencies of interictal neuromagnetic responses were in a normal level as compared with controls. These MEG results suggest that prolongations of brain responses are associated with the headache attack phase and these prolongations of brain responses do not persist within the pain free period.

The amplitudes of ictal neuromagnetic responses following finger movements were significantly increased as compared with the amplitudes of interictal neuromagnetic responses, as well as, with controls. This observation is slightly different from previous reports on childhood migraine, which showed a trend of increased amplitude during a headache attack (ictal) without statistical significance [28], [29]. This difference is likely due to the number of subjects in the present study, which is larger than that within previous reports [28], [29], and due to improvements in MEG analysis and procedures leading to better accuracy. MEG waveforms filtered with our new MEG methods (see Figure 1) are clearer, as compared with previous reports [28], [29]. One of the important findings of this study is that amplitudes of interictal neuromagnetic responses were in a normal level as compared with controls. It seems that aberrant ictal amplitudes returned to a normal level interictally. This is the first report showing interictal normalization of the amplitudes of neuromagnetic responses related to finger-movements.

The cerebral mechanism of the aforementioned normalization of neuromagnetic responses remains unclear. There is evidence that neuromagnetic activation changes with the ictal-interictal cycle of migraine [32]. It has been hypothesized that the dynamic variation of cortical abnormality in migraineurs during headache attack phase may reflect a change of serotonin transmission [54]. The ictal neuromagnetic alteration of motor cortex activation may reflect a transient cortical dysfunction. Once the migraine attack subsides, the functionality of the motor cortex may return to a level that is close to normal. Thus, there are neurophysiological changes associated with the subsidence of migraine headache attacks. We postulate that ictal cerebral dysfunction during a migraine is neurophysiologically reversible to a certain degree.

The measurements of neuromagnetic high-gamma oscillations have shown that spectral power of motor cortical activation during headache attack phase was significantly increased as compared to controls, which is consistent with previous MEG studies [10], [29]. Increased brain activation has been considered to be a result of cortical hyperexcitability [7], [55], [56]. Although the underlying mechanisms of increased activation in the primary motor cortex remain unclear, cortical excitability is the target of many new treatments [10]. MEG study of cortical excitability may play an important role in developing better and more effective therapeutic interventions for migraine in the future [57].

One of the most interesting findings is the decrease of neuromagnetic high-gamma oscillations (or hypo-activation) interictally as compared with controls. Although there are reports on interictal neuromagnetic activation in migraine [10], [32], the previous studies mainly focused on low-frequency brain waveforms in adult migraine. This study specifically focused on high-gamma oscillations in the motor cortex comparing ictal and interictal neurophysiology. This MEG data demonstrated that spectral power of interictal neuromagnetic high-gamma oscillations was significantly lower than that of the ictal neuromagnetic high-gamma oscillations and controls.

This observation is very interesting and important for several reasons. First, the MEG results suggest that migraineurs, while pain free, have distinct “neurophysiologically patterns”, compared to control subjects without a history of migraine. Second, pain free periods are associated with unique aberrant brain activation, which is different from ictal aberrant brain activation. Cortical functionality in migraineurs changes from hyper-activation to hypo-activation as migraine moves from the headache attack to the pain free period. This neuromagnetic fluctuation of high-gamma oscillations appears to serve as a very useful biomarker to investigate the periodic nature of migraine using MEG as a noninvasive tool.

The neurophysiological underpinnings of the fluctuation of high-gamma oscillations comparing attack and pain free period have rarely been studied with MEG. Previous reports have shown that repetitive transcranial magnetic stimulation (rTMS) can normalize habituation of the somatosensory system in migraine patients [58], [59]. It seems that dysfunctioning thalamo-cortical loops may be responsible for the interictal habituation deficit in migraine. It has also been found that about two-thirds (65%) of patients affected by either migraine type present an increased phosphene threshold in the interictal period, which suggests that their visual cortex is hypoexcitable during headache free phase [60]. A recent report using EEG has found that the amplitude of interictal early presynaptic high-frequency oscillations in the somatosensory system was significantly correlated to the clinical evolution [7]. Noteworthy, there is accumulating evidence that patients with migraine during pain free periods might be associated with cortical hypo-activation. The exact cerebral mechanism of the fluctuation of high-gamma oscillations in migraine remains unknown. Recent advances in neuroscience suggest that synaptic specialization turns interneuron networks into gamma frequency oscillators [61]. Specifically, the origin of high-gamma oscillations may be generated by GABAergic cortical interneurons. Consequently, the ictal hyper-activation and interictal hypo-activation may indicate an imbalance among excitatory and inhibitory cortical circuits that could predispose migraineurs to periodic headache attacks [7], [61].

Based on previous reports on migraine [28], [29], [53], we hypothesized that the source power of neuromagnetic high-gamma oscillations changes with the phases of periodic headache attack in childhood migraine. This study is clinically important for at least two reasons. First, our MEG data were recorded from children with migraine which might be unique as compared with many previous reports focusing on adult migraine [10], [32], [48]. Second, recent reports [62]–[71] have shown that normalization of cortical excitability may prevent and even cure migraine headaches. If the location and type of cortical dysfunction occurring during the phases of migraine attacks could be noninvasively determined, all of the preventions and treatments targeted at cortical dysfunction [39], [72], [73] – which currently show great promise – could be specified, refined, and their outcomes significantly improved in the future.

The results of MEG source localization have revealed that neuromagnetic high-gamma oscillations are well-localized. The present results are consistent with previous reports that show that MEG provides excellent localization accuracy especially for superficial sources [74], [75]. Papadelis and colleagues have found that the human high-frequency oscillations (HFOs) can be reliably detected noninvasively. A superficial phantom's source was localized with an accuracy of 2–3 mm with MEG. In addition, MEG sources can be linked with the cytoarchitectonic identity of the underlying region [74]. This observation is important and might be critical for developing spatially targeted treatments for migraine. For example, high-frequency rTMS increase and low-frequency rTMS decrease neural excitability of the stimulated cortex [40], [56], [76]. If neuromagnetic high-gamma oscillations could reliably reveal the location and types of cortical dysfunction occurring during the phases of migraine attacks, all of the preventions and treatments targeted at cortical excitability [39], [72], [73] could be refined and optimized. Specifically, the present study showed that the spectral power of neuromagnetic high-gamma oscillations was increased in children with migraine, during headache attack phase, while decreased in children with migraine, during headache free phase, in children with migraine. Building on previous results [74], [75] and the present data, noninvasive stimulation (e.g. TMS) may be used to spatially adjust cortical excitability during and between headache attacks, so as to cure and prevent headache attacks for migraine patients. Therefore, we consider the present study to lay an important foundation for clinical management of migraine in the future.

In conclusion, the MEG results of the present study have demonstrated that there was interictal normalization of motor cortical activation in a low-frequency range (1–50 Hz). However, the strength of interictal high-gamma oscillations (65–150 Hz) was significantly lower than that of ictal high-gamma oscillations, as well as, controls in the motor cortex. The ictal hyper-activation and interictal hypo-activation may indicate an imbalance among excitatory and inhibitory cortical circuits that could predispose migraineurs to periodic migraine attacks.

## References

[1] Hershey AD (2010) Recent developments in pediatric headache. Current opinion in neurology 23: 249–253. 20389244 10.1097/WCO.0b013e3283391888

[2] Lewis DW (2009) Pediatric migraine. Neurologic clinics 27: 481–501. 19289227 10.1016/j.ncl.2008.11.003

[3] Lipton RB, Pan J (2004) Is migraine a progressive brain disease? JAMA: the journal of the American Medical Association 291: 493. 14747508 10.1001/jama.291.4.493

[4] Turk WR (2000) Childhood migraine. Advances in pediatrics 47: 161–197. 10959443

[5] Young WB, Gangal KS, Aponte RJ, Kaiser RS (2007) Migraine with unilateral motor symptoms: a case-control study. Journal of neurology, neurosurgery, and psychiatry 78: 600–604. 10.1136/jnnp.2006.100214 PMC2077953 17056632

[6] Zach V, Bezov D, Lipton RB, Ashina S (2010) Headache associated with moyamoya disease: a case story and literature review. The journal of headache and pain 11: 79–82. 20012551 10.1007/s10194-009-0181-8 PMC3452187

[7] Restuccia D, Vollono C, Del Piero I, Martucci L, Zanini S (2012) Somatosensory High Frequency Oscillations reflect clinical fluctuations in migraine. Clinical neurophysiology: official journal of the International Federation of Clinical Neurophysiology 123: 2050–2056. 22554785 10.1016/j.clinph.2012.03.009

[8] Chen WT, Lin YY, Fuh JL, Hamalainen MS, Ko YC, et al (2011) Sustained visual cortex hyperexcitability in migraine with persistent visual aura. Brain: a journal of neurology 134: 2387–2395. 21729907 10.1093/brain/awr157

[9] Bramanti P, Grugno R, Vitetta A, Di Bella P, Muscara N, et al (2005) Migraine with and without aura: electrophysiological and functional neuroimaging evidence. Functional neurology 20: 29–32. 15948565

[10] Bowyer SM, Mason KM, Moran JE, Tepley N, Mitsias PD (2005) Cortical hyperexcitability in migraine patients before and after sodium valproate treatment. Journal of clinical neurophysiology: official publication of the American Electroencephalographic Society 22: 65–67. 15689715 10.1097/01.wnp.0000150928.23523.a9

[11] Vincent MB, Carvalho-e-Silva FM, Luiz RR (2011) The digiti quinti sign in hemiplegic migraine. Cephalalgia: an international journal of headache 31: 13–17. 20974599 10.1177/0333102410372424

[12] Toldo I, Cecchin D, Sartori S, Calderone M, Mardari R, et al (2011) Multimodal neuroimaging in a child with sporadic hemiplegic migraine: a contribution to understanding pathogenesis. Cephalalgia: an international journal of headache 31: 751–756. 21172953 10.1177/0333102410392068

[13] Kedia S, Stence N, Manco-Johnson M, Armstrong-Wells J, Bernard TJ (2012) Late cytotoxic edema in 2 children with hemiplegia: hemiplegic migraine or stroke? Headache 52: 674–678. 22404149 10.1111/j.1526-4610.2012.02116.x PMC3682476

[14] Jen JC (1993) Familial Hemiplegic Migraine. In: Pagon RA, Bird TD, Dolan CR, Stephens K, GeneReviews. Seattle (WA).

[15] Friberg L, Olsen TS, Roland PE, Lassen NA (1987) Focal ischaemia caused by instability of cerebrovascular tone during attacks of hemiplegic migraine. A regional cerebral blood flow study. Brain: a journal of neurology 110 (Pt 4) 917–934. 3651801 10.1093/brain/110.4.917

[16] Scherer P, Bauer H, Baum K (1997) Alternate finger tapping test in patients with migraine. Acta neurologica scandinavica 96: 392–396. 9449478 10.1111/j.1600-0404.1997.tb00304.x

[17] Esposito M, Verrotti A, Gimigliano F, Ruberto M, Agostinelli S, et al (2012) Motor coordination impairment and migraine in children: a new comorbidity? European journal of pediatrics 171: 1599–1604. 22673929 10.1007/s00431-012-1759-8

[18] Yu D, Yuan K, Zhao L, Zhao L, Dong M, et al (2012) Regional homogeneity abnormalities in patients with interictal migraine without aura: a resting-state study. NMR Biomed 25: 806–812. 22020869 10.1002/nbm.1796

[19] Maertens de Noordhout A, Pepin JL, Schoenen J, Delwaide PJ (1992) Percutaneous magnetic stimulation of the motor cortex in migraine. Electroencephalography and clinical neurophysiology 85: 110–115. 1373363 10.1016/0168-5597(92)90076-n

[20] Khedr EM, Ahmed MA, Mohamed KA (2006) Motor and visual cortical excitability in migraineurs patients with or without aura: transcranial magnetic stimulation. Neurophysiol Clin 36: 13–18. 16530139 10.1016/j.neucli.2006.01.007

[21] Fumal A, Bohotin V, Vandenheede M, Schoenen J (2003) Transcranial magnetic stimulation in migraine: a review of facts and controversies. Acta neurologica Belgica 103: 144–154. 14626694

[22] Conte A, Barbanti P, Frasca V, Iacovelli E, Gabriele M, et al (2010) Differences in short-term primary motor cortex synaptic potentiation as assessed by repetitive transcranial magnetic stimulation in migraine patients with and without aura. Pain 148: 43–48. 19854575 10.1016/j.pain.2009.09.031

[23] Conforto AB, Moraes MS, Amaro E Jr, Young WB, Lois LA, et al (2012) Increased variability of motor cortical excitability to transcranial magnetic stimulation in migraine: a new clue to an old enigma. J Headache Pain 13: 29–37. 21881905 10.1007/s10194-011-0379-4 PMC3253159

[24] Aurora SK, al-Sayeed F, Welch KM (1999) The cortical silent period is shortened in migraine with aura. Cephalalgia: an international journal of headache 19: 708–712. 10570724 10.1046/j.1468-2982.1999.019008708.x

[25] Brighina F, Palermo A, Daniele O, Aloisio A, Fierro B (2010) High-frequency transcranial magnetic stimulation on motor cortex of patients affected by migraine with aura: a way to restore normal cortical excitability? Cephalalgia: an international journal of headache 30: 46–52. 19438928 10.1111/j.1468-2982.2009.01870.x

[26] de Tommaso M, Brighina F, Fierro B, Francesco VD, Santostasi R, et al (2010) Effects of high-frequency repetitive transcranial magnetic stimulation of primary motor cortex on laser-evoked potentials in migraine. J Headache Pain 11: 505–512. 20714776 10.1007/s10194-010-0247-7 PMC3476225

[27] Xiang J, Degrauw X, Korostenskaja M, Korman AM, O'Brien HL, et al.. (2013) Altered Cortical Activation in Adolescents With Acute Migraine: A Magnetoencephalography Study. J Pain. 10.1016/j.jpain.2013.04.009 PMC3844550 23792072

[28] Guo X, Xiang J, Wang Y, O'Brien H, Kabbouche M, et al (2012) Aberrant neuromagnetic activation in the motor cortex in children with acute migraine: a magnetoencephalography study. PloS one 7: e50095. 23185541 10.1371/journal.pone.0050095 PMC3502360

[29] Wang X, Xiang J, Wang Y, Pardos M, Meng L, et al (2010) Identification of abnormal neuromagnetic signatures in the motor cortex of adolescent migraine. Headache 50: 1005–1016. 20487034 10.1111/j.1526-4610.2010.01674.x

[30] Mackert BM (2004) The discovery of slowness–recent progress in DC-MEG research. Neurology & clinical neurophysiology: NCN 2004: 41. 16012603

[31] Hall SD, Barnes GR, Hillebrand A, Furlong PL, Singh KD, et al (2004) Spatio-temporal imaging of cortical desynchronization in migraine visual aura: a magnetoencephalography case study. Headache 44: 204–208. 15012656 10.1111/j.1526-4610.2004.04048.x

[32] Chen WT, Wang SJ, Fuh JL, Lin CP, Ko YC, et al (2009) Peri-ictal normalization of visual cortex excitability in migraine: an MEG study. Cephalalgia: an international journal of headache 29: 1202–1211. 19558536 10.1111/j.1468-2982.2009.01857.x

[33] Bowyer SM, Aurora KS, Moran JE, Tepley N, Welch KM (2001) Magnetoencephalographic fields from patients with spontaneous and induced migraine aura. Annals of neurology 50: 582–587. 11706963 10.1002/ana.1293

[34] Huo X, Wang Y, Kotecha R, Kirtman EG, Fujiwara H, et al (2011) High-gamma oscillations of sensorimotor cortex during unilateral movement in the developing brain: a MEG study. Brain topography 23: 375–384. 20577795 10.1007/s10548-010-0151-0

[35] Magis D, Schoenen J (2011) Treatment of migraine: update on new therapies. Current opinion in neurology 24: 203–210. 21464715 10.1097/WCO.0b013e3283462c3f

[36] Ward TN (2010) Transcranial magnetic stimulation. Headache 50: 1629. 21198565 10.1111/j.1526-4610.2010.01786.x

[37] Lo YL (2010) Headache: migraine, magnetic stimulation and cortical excitability. Nature reviews Neurology 6: 425–427. 10.1038/nrneurol.2010.109 20689566

[38] Lipton RB, Pearlman SH (2010) Transcranial magnetic simulation in the treatment of migraine. Neurotherapeutics: the journal of the American Society for Experimental NeuroTherapeutics 7: 204–212. 20430320 10.1016/j.nurt.2010.03.002 PMC5084102

[39] Rapoport A (2011) New frontiers in headache therapy. Neurological sciences: official journal of the Italian Neurological Society and of the Italian Society of Clinical Neurophysiology 32 Suppl 1S105–109. 10.1007/s10072-011-0542-3 21533724

[40] Teepker M, Hotzel J, Timmesfeld N, Reis J, Mylius V, et al (2010) Low-frequency rTMS of the vertex in the prophylactic treatment of migraine. Cephalalgia: an international journal of headache 30: 137–144. 19515124 10.1111/j.1468-2982.2009.01911.x

[41] Siniatchkin M, Sendacki M, Moeller F, Wolff S, Jansen O, et al (2012) Abnormal changes of synaptic excitability in migraine with aura. Cereb Cortex 22: 2207–2216. 22079926 10.1093/cercor/bhr248

[42] Hershey AD (2005) Pediatric headache. Pediatric annals 34: 426–429. 16189894 10.3928/0090-4481-20050601-04

[43] Hershey AD (2010) Current approaches to the diagnosis and management of paediatric migraine. Lancet neurology 9: 190–204. 20129168 10.1016/S1474-4422(09)70303-5

[44] Wang Y, Xiang J, Vannest J, Holroyd T, Narmoneva D, et al (2011) Neuromagnetic measures of word processing in bilinguals and monolinguals. Clinical neurophysiology: official journal of the International Federation of Clinical Neurophysiology 122: 1706–1717. 21414839 10.1016/j.clinph.2011.02.008

[45] Huo X, Xiang J, Wang Y, Kirtman EG, Kotecha R, et al (2010) Gamma oscillations in the primary motor cortex studied with MEG. Brain & development 32: 619–624. 19836911 10.1016/j.braindev.2009.09.021

[46] Korostenskaja M, Pardos M, Fujiwara H, Kujala T, Horn P, et al (2010) Neuromagnetic evidence of impaired cortical auditory processing in pediatric intractable epilepsy. Epilepsy research 92: 63–73. 20863661 10.1016/j.eplepsyres.2010.08.008

[47] Xiang J, Wang Y, Chen Y, Liu Y, Kotecha R, et al (2010) Noninvasive localization of epileptogenic zones with ictal high-frequency neuromagnetic signals. Journal of neurosurgery Pediatrics 5: 113–122. 20043746 10.3171/2009.8.PEDS09345

[48] Bowyer SM, Tepley N, Papuashvili N, Kato S, Barkley GL, et al (1999) Analysis of MEG signals of spreading cortical depression with propagation constrained to a rectangular cortical strip. II. Gyrencephalic swine model. Brain research 843: 79–86. 10528113 10.1016/s0006-8993(99)01893-4

[49] Society HCCotIH (1988) Classification and diagnostic criteria for headache disorders, cranial neuralgias and facial pain. Cephalalgia: an international journal of headache 8 Suppl 71–96. 3048700

[50] Hershey AD, Winner P, Kabbouche MA, Gladstein J, Yonker M, et al (2005) Use of the ICHD-II criteria in the diagnosis of pediatric migraine. Headache 45: 1288–1297. 16324160 10.1111/j.1526-4610.2005.00260.x

[51] Wilke M, Holland SK, Altaye M, Gaser C (2008) Template-O-Matic: a toolbox for creating customized pediatric templates. NeuroImage 41: 903–913. 18424084 10.1016/j.neuroimage.2008.02.056

[52] Yoshino A, Okamoto Y, Onoda K, Shishida K, Yoshimura S, et al (2012) Sadness enhances the experience of pain and affects pain-evoked cortical activities: an MEG study. J Pain 13: 628–635. 22515946 10.1016/j.jpain.2011.12.005

[53] Korostenskaja M, Pardos M, Kujala T, Rose DF, Brown D, et al (2011) Impaired auditory information processing during acute migraine: a magnetoencephalography study. The International journal of neuroscience 121: 355–365. 21425948 10.3109/00207454.2011.560312

[54] Sakai Y, Dobson C, Diksic M, Aube M, Hamel E (2008) Sumatriptan normalizes the migraine attack-related increase in brain serotonin synthesis. Neurology 70: 431–439. 18250288 10.1212/01.wnl.0000299095.65331.6f

[55] Chen WT, Wang SJ, Fuh JL, Ko YC, Lee YC, et al (2012) Visual cortex excitability and plasticity associated with remission from chronic to episodic migraine. Cephalalgia: an international journal of headache 32: 537–543. 22529191 10.1177/0333102412443337

[56] Brigo F, Storti M, Nardone R, Fiaschi A, Bongiovanni LG, et al (2012) Transcranial magnetic stimulation of visual cortex in migraine patients: a systematic review with meta-analysis. J Headache Pain 13: 339–349. 22535147 10.1007/s10194-012-0445-6 PMC3381069

[57] Hershey AD, Kabbouche MA, Powers SW (2010) Treatment of pediatric and adolescent migraine. Pediatric annals 39: 416–423. 20666347 10.3928/00904481-20100623-06

[58] Coppola G, De Pasqua V, Pierelli F, Schoenen J (2012) Effects of repetitive transcranial magnetic stimulation on somatosensory evoked potentials and high frequency oscillations in migraine. Cephalalgia: an international journal of headache 32: 700–709. 22652384 10.1177/0333102412446313

[59] Coppola G, Di Clemente L, Fumal A, Magis D, De Pasqua V, et al (2007) Inhibition of the nociceptive R2 blink reflex after supraorbital or index finger stimulation is normal in migraine without aura between attacks. Cephalalgia: an international journal of headache 27: 803–808. 17598762 10.1111/j.1468-2982.2007.01323.x

[60] Bohotin V, Fumal A, Vandenheede M, Bohotin C, Schoenen J (2003) Excitability of visual V1–V2 and motor cortices to single transcranial magnetic stimuli in migraine: a reappraisal using a figure-of-eight coil. Cephalalgia: an international journal of headache 23: 264–270. 12716343 10.1046/j.1468-2982.2003.00475.x

[61] Bartos M, Vida I, Jonas P (2007) Synaptic mechanisms of synchronized gamma oscillations in inhibitory interneuron networks. Nat Rev Neurosci 8: 45–56. 17180162 10.1038/nrn2044

[62] Bianchin MM, Londero RG, Lima JE, Bigal ME (2010) Migraine and epilepsy: a focus on overlapping clinical, pathophysiological, molecular, and therapeutic aspects. Current pain and headache reports 14: 276–283. 20495966 10.1007/s11916-010-0121-y

[63] Clarke T, Baskurt Z, Strug LJ, Pal DK (2009) Evidence of shared genetic risk factors for migraine and rolandic epilepsy. Epilepsia 50: 2428–2433. 19674062 10.1111/j.1528-1167.2009.02240.x

[64] Caraballo R, Koutroumanidis M, Panayiotopoulos CP, Fejerman N (2009) Idiopathic childhood occipital epilepsy of Gastaut: a review and differentiation from migraine and other epilepsies. Journal of child neurology 24: 1536–1542. 19955346 10.1177/0883073809332395

[65] Rogawski MA (2008) Common pathophysiologic mechanisms in migraine and epilepsy. Archives of neurology 65: 709–714. 18541791 10.1001/archneur.65.6.709

[66] Piccinelli P, Borgatti R, Nicoli F, Calcagno P, Bassi MT, et al (2006) Relationship between migraine and epilepsy in pediatric age. Headache 46: 413–421. 16618257 10.1111/j.1526-4610.2006.00373.x

[67] Haut SR, Bigal ME, Lipton RB (2006) Chronic disorders with episodic manifestations: focus on epilepsy and migraine. Lancet neurology 5: 148–157. 16426991 10.1016/S1474-4422(06)70348-9 PMC1457022

[68] Mauskop A (2005) Vagus nerve stimulation relieves chronic refractory migraine and cluster headaches. Cephalalgia: an international journal of headache 25: 82–86. 15658944 10.1111/j.1468-2982.2005.00611.x

[69] Sankar R (2004) Initial treatment of epilepsy with antiepileptic drugs: pediatric issues. Neurology 63: S30–39. 15557549 10.1212/wnl.63.10_suppl_4.s30

[70] Sand T (2003) Electroencephalography in migraine: a review with focus on quantitative electroencephalography and the migraine vs. epilepsy relationship. Cephalalgia: an international journal of headache 23 Suppl 15–11. 12699455 10.1046/j.1468-2982.2003.00570.x

[71] Bigal ME, Lipton RB, Cohen J, Silberstein SD (2003) Epilepsy and migraine. Epilepsy & behavior: E&B 4 Suppl 2S13–24. 10.1016/j.yebeh.2003.07.003 14527480

[72] Coppola G, Schoenen J (2012) Cortical excitability in chronic migraine. Current pain and headache reports 16: 93–100. 22076672 10.1007/s11916-011-0231-1

[73] Bigal ME, Serrano D, Buse D, Scher A, Stewart WF, et al (2008) Acute migraine medications and evolution from episodic to chronic migraine: a longitudinal population-based study. Headache 48: 1157–1168. 18808500 10.1111/j.1526-4610.2008.01217.x

[74] Papadelis C, Eickhoff SB, Zilles K, Ioannides AA (2011) BA3b and BA1 activate in a serial fashion after median nerve stimulation: direct evidence from combining source analysis of evoked fields and cytoarchitectonic probabilistic maps. NeuroImage 54: 60–73. 20691793 10.1016/j.neuroimage.2010.07.054 PMC8015308

[75] Papadelis C, Poghosyan V, Fenwick PB, Ioannides AA (2009) MEG's ability to localise accurately weak transient neural sources. Clinical neurophysiology: official journal of the International Federation of Clinical Neurophysiology 120: 1958–1970. 19782641 10.1016/j.clinph.2009.08.018

[76] Minks E, Kopickova M, Marecek R, Streitova H, Bares M (2010) Transcranial magnetic stimulation of the cerebellum. Biomed Pap Med Fac Univ Palacky Olomouc Czech Repub 154: 133–139. 20668494 10.5507/bp.2010.020

